# Regulatory effects of smoking cessation on the cellular microenvironment and differentially expressed genes in precancerous lesions of pulmonary nodules in mice based on single-cell RNA sequencing and immune repertoire-sequencing

**DOI:** 10.1371/journal.pone.0356148

**Published:** 2026-08-13

**Authors:** Xintong Wang, Fang Tang, Jiayu Qin, Tiquan Xiao, Liwei Shi, Shujun Zhang, Chunli Che

**Affiliations:** 1 Department of Respiratory and Critical Care Medicine, The Fourth Affiliated Hospital of Harbin Medical University, Harbin, China; 2 Harbin Institute of Technology, Harbin, China; 3 Department of Pathology, The Fourth Affiliated Hospital of Harbin Medical University, Harbin, China; Xiangya Hospital Central South University, CHINA

## Abstract

**Background:**

Smoking cessation decreases lung cancer progression; however, its effects on precancerous lesions and the underlying mechanisms remain unclear. This study established a mouse model of precancerous pulmonary nodules and employed single-cell RNA sequencing (scRNA-seq) and immune repertoire sequencing (IR-seq) to elucidate the regulatory mechanisms by which smoking cessation influences the development of lung precancerous lesions.

**Methods:**

A B[a]P-induced mouse model of pulmonary precancerous lesions was established after 6 weeks of B[a]P treatment. Lung nodules were identified via micro-MRI, and histopathological features were assessed by H&E staining. After confirming the establishment of precancerous lesions in smoking-exposed mice, graded B[a]P withdrawal for 4 and 8 weeks was implemented. Nodule size was measured using Generic Medical Imaging software. scRNA-seq and IR-seq analyzed immune cell composition, proportions, signaling, and gene expression.

**Results:**

B[a]P withdrawal reduced new lung nodule formation and decreased the diameter, area, and volume of existing nodules. It alleviated cellular atypia, reduced inflammation, and altered the tumor microenvironment by increasing T-, B-, and NK cells while reducing monocytes and neutrophils. Concurrently, B[a]P withdrawal downregulated Vim, GPX1, Atf3, CD44, and Arpc5 and upregulated Hsph1, Hsp90aa, and Hspa1b. B[a]P withdrawal upregulated CD80 and ICOS, suppressed intercellular communication via Icam1-(Itgal+Itgb2), Ppia-Bsg, and Thbs1-Cd47 pathways. The APP-CD74 pathway emerged as a novel regulator in precancerous lesions. IR-seq revealed restored T- and B-cell receptor diversity, CDR3 sequence alterations, and V(D)J gene regulation.

**Conclusions:**

B[a]P withdrawal reduces lung nodules, inhibits progression, potentially by enhancing immune surveillance by modulating immune cells, signaling pathways, and gene expression, while promoting ferroptosis, suppressing inflammation, and preventing tumor stem cell-like traits. These findings may provide mechanistic insights into the beneficial effects associated with smoking cessation.

## 1. Introduction

Lung cancer development associated with smoking follows a prolonged disease progression. Precancerous lesions represent a critical pathological stage in lung cancer onset, with epithelial–mesenchymal transition (EMT) and cancer stem cell (CSC) formation being key processes in these lesions [1]. These lesions are increasingly recognized as biologically dynamic rather than static, and their cellular plasticity may influence subsequent disease progression. Tobacco contains various carcinogenic substances, including polycyclic aromatic hydrocarbons-benzo[a]pyrene (PAH-B[a]P), which interacts with cytochrome P450 monooxygenase enzyme 1 (CYP1A1), a member of the cytochrome P450 superfamily in bronchial and alveolar epithelial cells. This interaction induces missense mutations in CYP1A1 DNA, activating oncogenic signaling [2]. Furthermore, PAH-B[a]P initiates the tumor growth factor-beta (TGF-β) and Snail signaling pathways by increasing reactive oxygen species production, leading to the loss of proliferation inhibition in alveolar type II epithelial (AT2) cells and the subsequent initiation of EMT [3]. Under prolonged exposure to carcinogens such as PAH-B[a]P, AT2 cells in the EMT state lose polarity, exhibit nuclear instability and atypia, and acquire CSC-like phenotypes, progressing into precancerous lesions. Despite the established role of smoking in the early stage of lung cancer development, whether smoking cessation suppresses malignant progression or induces benign outcomes remains unknown.

Smoking cessation after a lung cancer diagnosis can reduce disease progression and mortality, improve the 5-year overall survival rate, and markedly enhance progression-free survival for patients with early-stage lung cancer [4]. However, whether smoking cessation reduces the incidence of lung cancer or the occurrence and progression of precancerous lesions is unclear. In this context, emerging evidence suggests that the local microenvironment, particularly the immune microenvironment, may play a role in determining the fate of premalignant lesions. Chronic exposure to tobacco carcinogens has been associated with persistent inflammation and disruption of immune homeostasis, which may contribute to lesion persistence and progression. Conversely, smoking cessation may partially restore immune balance and influence interactions between epithelial and immune cells. However, the cellular composition, intercellular communication patterns, and underlying molecular features associated with these changes remain incompletely understood.

Therefore, a clearer understanding of how smoking cessation influences lung precancerous lesions is warranted. In particular, defining lesion-associated cellular and molecular characteristics, as well as exploring potential biomarkers related to lesion progression or stabilization, may help to better characterize early lung carcinogenesis and inform strategies aimed at reducing the risk of progression to invasive lung cancer.

## 2. Methods

### 2.1. Establishment of a mouse model of pulmonary precancerous lesions

All experimental protocols were approved by the Animal Welfare Committee of Harbin Medical University (approval number: 2024-DWSYLLCZ-29) and conducted in accordance with institutional guidelines and the National Guidelines for Ethical Review of Laboratory Animal Welfare (GB/T 35892−2018), China. This study complies with the ARRIVE guidelines. Five-week-old female Kunming mice were obtained from Changsheng Biotechnology Co., Ltd. (License No.: SCXK 2020−0001; Liaoning, China). The mice were divided into groups A, B, C, and D (n = 32 per group). All mice were housed in a specific pathogen-free environment and weighed weekly. Daily monitoring was performed by trained personnel to assess appearance, posture, grooming, activity, food and water intake, and signs of respiratory or systemic distress. Predefined humane endpoints included ≥20% body-weight loss from baseline; persistent severe dyspnea, cyanosis, or labored breathing; inability to eat or drink for more than 48 hours; severe lethargy or impaired mobility; signs of infection, ulceration, or uncontrolled bleeding; or any condition judged by the attending veterinarian to indicate undue pain or suffering. Animals meeting these criteria were to be euthanized via isoflurane overdose followed by cervical dislocation in accordance with institutional and AVMA guidelines. No animals reached humane endpoints. During the study, four animals in Group A and three animals in each of Groups B, C, and D died during routine observation of causes not meeting humane endpoint criteria, resulting in 28 surviving mice in Group A and 29 in Groups B, C, and D at the planned endpoint.

A B[a]P exposure model was established by oral gavage of B[a]P (purity≥96%; McLean Biochemical Technology, Shanghai, China), based on previous B[a]P-induced murine lung carcinogenesis studies [5–10], and was used here as a representative model of smoking-related carcinogenic exposure [11–13]. To investigate post-exposure changes in this B[a]P-based model, a smoking cessation-like intervention was simulated by discontinuing B[a]P administration after the exposure period. Group A (B[a]P exposure group) received intragastric administration of 3 µmol of B[a]P (dissolved in 0.1 mL olive oil) once per week for 6 consecutive weeks. Group B (4-week B[a]P withdrawal group) received the same B[a]P treatment as group A for 6 weeks, followed by B[a]P withdrawal for 4 weeks. Group C (8-week B[a]P withdrawal group) received the same B[a]P treatment as group A for 6 weeks, followed by B[a]P withdrawal for 8 weeks. Group D (vehicle control group) received intragastric administration of 0.1 mL olive oil once weekly for 6 consecutive weeks. The 4-week and 8-week withdrawal periods were selected as predefined post-exposure observation windows to assess the temporal trajectory of lung tissue changes following B[a]P withdrawal and to represent shorter and longer withdrawal intervals in this B[a]P-based model [14,15]. At the end of the experiment, the mice were anesthetized using a mixture of oxygen and isoflurane for MRI. Lung scans were performed using a 9.4 T MRI scanner (BioSpec 94/20 USR, Bruker, Germany) with a Turbo Spin Echo (TSE) sequence. Imaging parameters included TR 819.257 ms, TE 12 ms, matrix 256 × 256, FOV 38.4 × 38.4 mm², slice thickness 1.0 mm, and 15 slices. The respiratory rate and body temperature of the mice were monitored using electrocardiophysiological equipment during the MRI scan. After scanning, Generic Medical Imaging (GMI) software was used to label and measure lung nodules, followed by artificial intelligence (AI)-based regression validation and optimization of measurements.

**Fig 1:**
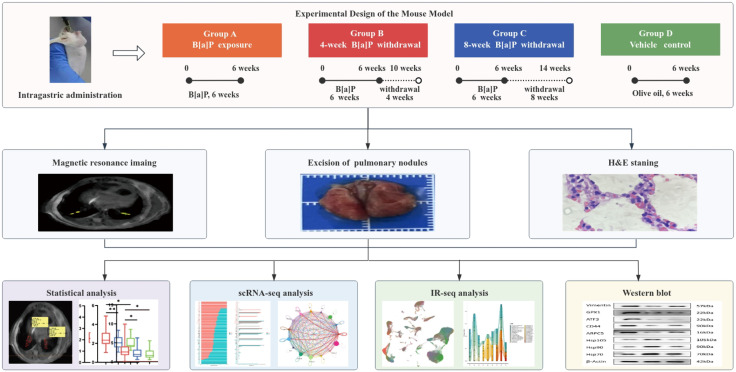
Overall technical route map of the experiment.

Pulmonary precancerous lesions were considered successfully established based on combined radiological and histopathological criteria. Radiologically, eligible lesions were defined as small, well-demarcated pulmonary nodules with limited size and number, without evidence of invasive growth, bronchial obstruction, pleural involvement, or distant metastasis. Histopathologically, hematoxylin and eosin (H&E)-stained sections were examined for features consistent with precancerous lesions, including alveolar epithelial hyperplasia predominantly involving type II alveolar epithelial (AT2) cells, an increased nuclear-to-cytoplasmic ratio, nuclear enlargement, multinucleation, and focal cellular atypia. Lesions showing histological features of invasive lung cancer, such as stromal invasion, vascular invasion, marked destruction of alveolar architecture, or solid tumor mass formation, were excluded. Only lesions that met both the radiological and histopathological criteria were included in the subsequent analyses. Mice were euthanized through cervical dislocation, and lung tissues with nodules were isolated for subsequent H&E staining (Yili Fine Chemicals Co., Ltd., Beijing, China), scRNA-seq, IR-seq and bioinformatics analysis (Fig 1).

### 2.2. Annotation and measurement of precancerous lung nodules

GMI software was used for lung nodule annotation and measurement [16]. DICOM files were imported after configuring the software (requirements include a specific processor, GPU, memory, hard drive space, and display resolution). The user manually traced the nodule boundaries, which were then automatically captured. Missing areas within the nodules were corrected using the “Multi-Layer Edit” command, and unconnected regions were segmented using the “Region Growth” command. The annotated and segmented nodule images were analyzed with GMI software to calculate diameter, area, volume, and density. AI was used to validate and optimize the GMI-generated annotations and measurements. The code used for this process was debugged and executed in Python 3.10.11.

### 2.3. H&E staining

Lung nodule tissues marked by MRI were fixed in 4% paraformaldehyde, embedded in paraffin, and sectioned into 3 μm slices. The slices were deparaffinized and dehydrated, followed by staining with hematoxylin for 7 min and differentiation in 1% hydrochloric acid ethanol for 3 s. Eosin was used to stain the cytoplasm for 3 min. Stained sections were imaged using a fluorescence-inverted microscope (Leica, Germany). These images were analyzed to determine whether the tissues exhibited precancerous lesions. The pathological morphology of precancerous cells after different durations of B[a]P withdrawal was compared to assess its effects on precancerous lesion progression or regression.

### 2.4. scRNA-seq, quality control, enrichment, and CellChat intercellular communication analysis

Fresh lung nodule tissue samples were collected from three mice per group, yielding nine samples, with each sample containing at least one lung nodule. scRNA-seq was performed using the BD Rhapsody system. The raw sequencing data were filtered based on the following criteria: number of genes between 400 and 5000, Unique Molecular Identifier count between 600 and 20000, and mitochondrial gene proportion < 20%. Principal Component Analysis was used for dimensionality reduction and significant principal component identification. Cell clustering analysis was conducted to identify cell subpopulations. Seurat’s FindMarkers function was used to identify DEGs across 15 cell types in groups A, B, and C, followed by Gene Ontology (GO) analysis to assess the enrichment of these DEGs in biological processes (BP). Finally, a cellular communication network was constructed using CellChat to evaluate the functional roles and importance of various cell types. The intercellular communication patterns were analyzed to determine how different cell types interact under different B[a]P withdrawal conditions.

### 2.5. IR-seq analysis

scRepertoire (v2.0.0) software was used to extract CDR3 sequences and perform clonal typing to analyze the rearrangement patterns of V, D, and J gene segments. Immune repertoire diversity was calculated using the Shannon and inverse Simpson diversity indices to compare changes in immune diversity between B[a]P exposure and withdrawal samples. The spatial distribution of TCR/BCR clones of varying sizes was analyzed to determine the distribution of immune cells across different samples and microenvironments. The dimensionality reduction of single-cell transcriptomic data was conducted using Seurat. The TCR/BCR data from scRepertoire were integrated with transcriptomic data to investigate the transcriptional features of specific immune clones. This integrated analysis identified associations between immune cell expansion types and their gene expression profiles, revealing differences in gene regulation within the immune microenvironment.

### 2.6. Statistical analyses

All statistical analyses were performed using GraphPad Prism 9.5 (GraphPad Software, Inc., La Jolla, CA, USA). Data are presented as means ± standard deviation or as medians (25th percentile, 75th percentile). A two-way repeated-measures analysis of variance (ANOVA) followed by multiple comparisons and the non-parametric Kruskal–Wallis test were used to compare differences between groups. A P-value of < 0.05 was considered statistically significant.

### 2.7. Western blot analysis

Lung tissue samples from each group were used for protein-level validation. Total protein was extracted using RIPA lysis buffer supplemented with protease inhibitors, and protein concentration was determined using a bicinchoninic acid (BCA) assay. Equal amounts of protein were separated by SDS–PAGE and transferred onto polyvinylidene fluoride (PVDF) membranes. After blocking with 5% non-fat milk at room temperature, membranes were incubated overnight at 4°C with primary antibodies against selected target proteins, followed by incubation with appropriate horseradish peroxidase (HRP)-conjugated secondary antibodies. Protein bands were visualized using an enhanced chemiluminescence (ECL) detection system. β-Actin was used as the loading control. All experiments were performed in triplicate.

## 3. Results

### 3.1. Comparative outcomes of lung premalignant lesion progression across different durations of B[a]P withdrawal

**Fig 2:**
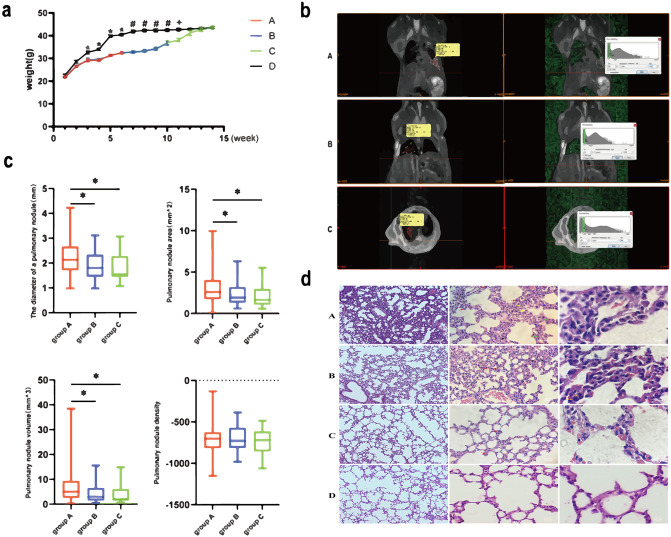
Lung nodule precancerous lesions in mice in the B[a]P exposure and B[a]P withdrawal groups. a, Line chart of body weight changes in mice after gradient B[a]P withdrawa. Data are presented as mean ± SD. Differences among groups were analyzed using two-way repeated-measures ANOVA followed by multiple comparisons. *P < 0.05 compared with group D versus groups A, B, and C; #P < 0.05 compared with group D versus groups B and C; + P < 0.05 compared with group D versus group C. Symbols indicate statistically significant differences at the corresponding time points. b, Generic Medical Imaging (GMI) software results for lung nodule annotation and measurement for groups A, B, and C, and the artificial intelligence (AI) regression verification results. c, Statistical analysis of lung nodule diameter, area, volume, and average density for groups A, B, and C; *, P < 0.05; **, P < 0.005. d, Hematoxylin–eosin (H&E)-stained histological sections of lung nodules from groups A, B, and C at magnifications of 100, 400, and 1000x. Key histopathological features are highlighted using colored arrows: red arrows indicate type II alveolar epithelial (AT2) cell hyperplasia; orange arrows indicate atypical AT2 cells; black arrows indicate inflammatory cell infiltration; yellow arrows indicate foam cell aggregation; green arrows indicate carbon particle deposition; and blue arrows indicate fibroblast proliferation.

During the early stage of the experiment, no significant differences in body weight were observed among the groups. Over time, the weight gain rate of mice exposed to B[a]P was lower than that of the healthy control group (group D). After B[a]P withdrawal, body weight gain gradually recovered, with more pronounced improvement observed as the duration of withdrawal increased (Fig 2a). Lung MRI examinations revealed that, among the surviving mice, 15 lung nodules were identified in Group A (28 survivors), whereas Groups B and C each had 29 surviving mice, with nine and seven lung nodules identified, respectively. No nodules were detected in Group D, in which 29 mice survived. Therefore, no statistical analysis of nodule incidence was performed. Compared to group A, the average diameter, area, and volume of nodules in groups B and C significantly reduced (P < 0.05, Fig 2b). The average density signal values also changed but were not significant (P = 0.69, Table 1, Fig 2c).Histopathological examination of hematoxylin–eosin (H&E)-stained lung sections revealed distinct morphological alterations among groups. In group A (B[a]P exposure group), lung nodule tissues exhibited partial alveolar cavity expansion, alveolar septal destruction, and carbon particle deposition within alveolar cavities and septa(green arrows). Mild inflammatory cell infiltration(black arrows), significant foam cell aggregation(yellow arrows), focal fibroblast proliferation with mild fibrosis(blue arrows), and alveolar epithelial hyperplasia (primarily AT2 cells) were observed(red arrows). Cells showed increased volume, nuclear enlargement, multinucleation, nuclear fragmentation, and substantial atypia(orange arrows). In group B (4-week B[a]P withdrawal group), mild inflammatory cell infiltration (black arrows) and focal alveolar cell hyperplasia were observed(red arrows), along with atypical epithelial cells, including abnormal AT2 cells(orange arrows). In group C (8-week B[a]P withdrawal group), focal alveolar cell proliferation was less pronounced(red arrows), with reduced cellular atypia compared to group B. In contrast, group D (vehicle control group) exhibited normal alveolar architecture, characterized by thin and intact alveolar septa, uniform alveolar spaces, and the absence of inflammatory cell infiltration, epithelial hyperplasia, or cellular atypia.These results confirm that shortening the duration of B[a]P administration established a mouse model of lung precancerous lesions. Additionally, B[a]P withdrawal, used here to mimic smoking cessation, alleviated inflammatory cell infiltration, reduced the atypia of AT2 cells, and mitigated fibroblast proliferation and fibrosis in precancerous tissues (Fig 2d).

**Table 1:** Comparison of lung nodule incidence and GMI software lung nodule calculation results.

group	Incidence of lung nodules (%)	Diameter of lung nodule (mm)	Lung nodule area (mm^2)	Lung nodule volume (mm^3)	Average signal value of lung nodule density
A	48.3%(15/28)	2.13(1.73, 2.63)	2.58(1.78, 3.95)	5.02(2.66, 9.13)	−705(−809.75, -627.88)
B	29.0%(9/29)^a^	1.79(1.47,2.30)^a^	1.92(1.33, 3.17)^a^	2.93(1.66, 6.33)^a^	−730(−812.75, -584.75)
C	22.6%(7/29)^a^	1.55(1.49, 2.20)^a^	1.64(1.17, 2.83)^a^	1.90(1.68, 5.53)^a^	−720.25(−844.125, -645.88)
p-value	0.0542	0.0039	0.0175	0.0047	0.6900

*Note: ᵃ Indicates a significant difference compared to Group A (p < 0.05). For the comparison among the three groups, the data do not meet the normal distribution assumption, so the Kruskal-Wallis test was used, and the median (25th percentile value, 75th percentile value) is presented. n = 32 for each group.*

### 3.2. Cell types and proportions in premalignant lesions during B[a]P exposure and after B[a]P withdrawal

**Fig 3:**
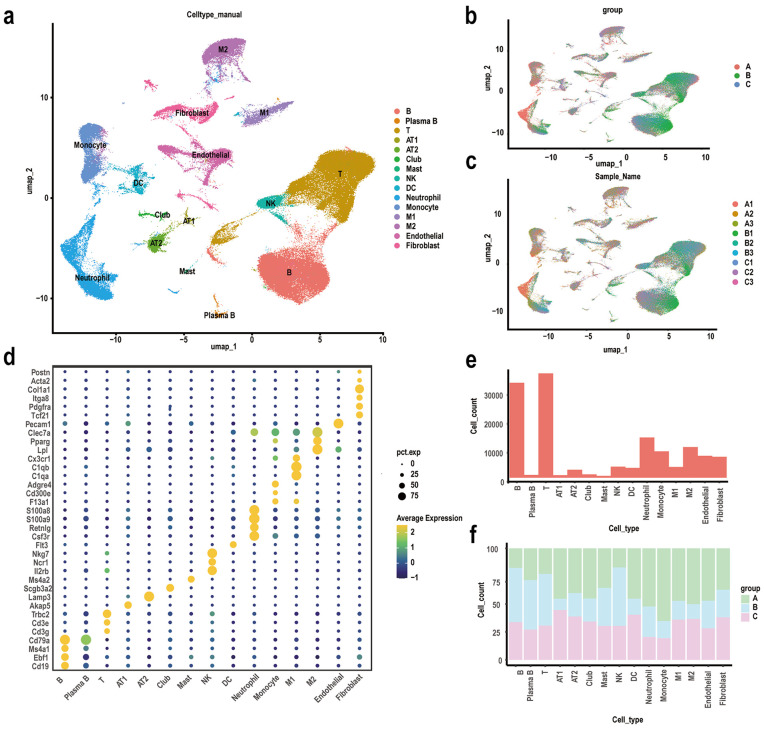
Single-cell RNA sequencing, cell types, and proportions in precancerous lung nodules in the B[a]P exposure and B[a]P withdrawal groups. a, t-SNE plot of single-cell RNA sequencing (scRNA-seq) data from precancerous lung nodule tissues of groups A, B, and C, where each dot represents a cell, and the color indicates cell type. b, c, Uniform Manifold Approximation and Projection (UMAP) of the proportion of 15 cell types in precancerous lung nodule tissues of groups A, B, and C, showing no batch effect. d, Dot plot showing the expression level distribution of marker genes for 15 cell types in precancerous lung nodule tissues of groups A, B, and C, where the x-axis corresponds to different cell subgroups, the y-axis corresponds to cell marker genes, the color of the dots represents the average expression level of the gene, and the size of the dots corresponds to the proportion of cells expressing the gene. e, Bar chart of the total number of different cell types in precancerous lung nodule tissues of groups A, B, and C. f, Bar chart of the proportion of different cell types in precancerous lung nodule tissues of groups A, B, and C.

After quality control and removing the batch effect, an aggregate gene expression matrix with 52,382 cells and 30,097 genes was generated. Sixty-five distinctive cell clusters were obtained with a resolution of 2.0 and visualized using a Uniform Manifold Approximation and Projection (UMAP) plot (S1 Fig). Each cluster was annotated using the cluster-specific marker genes to identify the primary cell types (S2 Fig). Unsupervised clustering of gene expression profiles identified marker genes for cell subclusters, enabling annotation of 15 cell types across all groups (Fig 3a–d), including B cells (Cd19, Ebf1, Ms4a1, Cd79a); plasma B cells (Cd19a + , Cd19-); T-cells (Cd3g, Cd3e, Trbc2); AT1 cells (Akap5); AT2 cells (Lamp3); club cells (Scgb3a2); mast cells (Ms4a2); natural killer (NK) cells (Il2rb, Ncr1, Nkg7); dentritic cells (Flt3); neutrophils (Csf3r, Retnlg, S100a9, S100a8); monocytes (F13a1, Cd300e, Adgre4); M1 macrophages (C1qa, C1qb, Cx3cr1); M2 macrophages (Lpl, Pparg, Clec7a); endothelial cells (Pecam1); and fibroblasts (Tcf21, Pdgfra, Itga8, Col1a1, Acta2, Postn). Quantitative analysis of cell type proportions revealed significant differences among the three groups (Fig 3e–f). The B[a]P exposure group (group A) showed a markedly higher proportion of monocytes (inflammatory cells) with a lower proportion of B, T, and NK cells (immune response cells) than the B[a]P withdrawa groups (groups B and C). These findings indicate that B[a]P withdrawa alters the cellular composition of the precancerous lesion microenvironment by reducing the proportion of inflammation-associated cells and increasing immune response-associated cells.

### 3.3. DEG identification and enrichment analysis in premalignant lesions pduring B[a]P exposure and after B[a]P withdrawal

**Fig 4:**
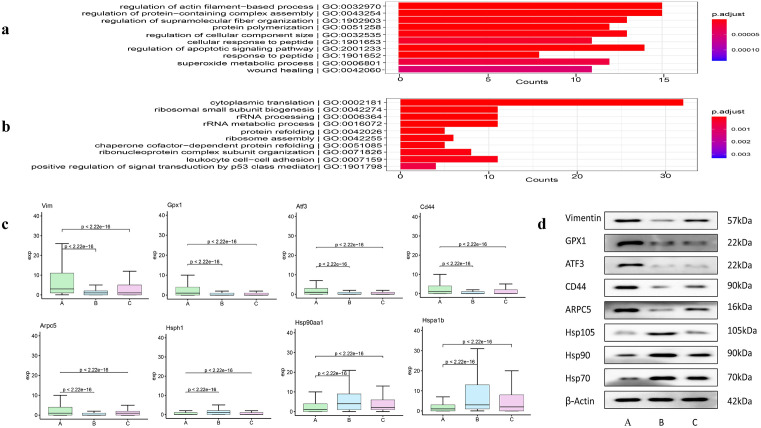
Differential gene screening and enrichment analysis in precancerous lesions in the B[a]P exposure and B[a]P withdrawal groups. a, Bar chart of Gene Ontology (GO) enrichment analysis for upregulated genes in the biological process (BP) results between the B[a]P exposure and B[a]P withdrawal groups, showing the top 10 terms with the smallest p.adjust values, where the x-axis represents the number of genes enriched in the pathways. b, Bar chart of GO enrichment analysis for downregulated genes in the BP results between the B[a]P exposure and B[a]P withdrawal groups, showing the top 10 terms with the smallest p.adjust values, where the x-axis represents the number of genes enriched in the pathways. c, Box plot of the expression levels of differential genes between the B[a]P exposure and B[a]P withdrawal groups in A, B, and C.d: Western blot validation of representative genes with the highest fold enrichment in the enriched biological processes; protein levels of Vimentin (Vim), GPX1, ATF3, CD44, and ARPC5 were higher in the B[a]P exposure group, whereas Hsp105 (Hsph1), Hsp90 (Hsp90aa), and Hsp70 (Hspa1b) were lower in the B[a]P exposure group compared with the post-exposure groups; β-Actin was used as the loading control.

Compared with the B[a]P withdrawal groups, the B[a]P exposure group displayed significantly upregulated BP, including regulation of actin filament-based processes (FDR: 1.76 × 10−10), regulation of protein complex assembly (FDR: 8.15 × 10^−10^), and regulation of supramolecular fiber organization (FDR: 1.97 × 10^−9^) (Fig 4a, b; S1 Table). The top five DEGs with the highest fold enrichment were *Vim*, *GPX1*, *Atf3*, *CD44*, and *Arpc5* (Fig 4c; S3 Fig). These findings suggest that B[a]P exposure promotes precancerous lesion development by enhancing EMT and CSC-associated features [17–20]. Vim, CD44, and Arpc5 may represent candidate regulatory genes involved in this transition [17,19,20]. Additionally, the significant upregulation of *GPX1* in precancerous tissues from the B[a]P exposure group corroborates the inhibition of ferroptosis in tumor cells [21]. This observation highlights the early stages of B[a]P-induced lung carcinogenesis, where *GPX1* expression is upregulated to suppress ferroptosis and facilitate the formation of precancerous lesions. Compared with the B[a]P withdrawal groups, the most significantly downregulated BP were cytoplasmic translation (FDR: 8.59 × 10^−44^), ribosomal small subunit biogenesis (FDR: 1.39 × 10  ^−11^), and rRNA processing (FDR: 4.62 × 10 ^−6^) (Fig 4b; S1 Table). Additionally, the positive regulation of signal transduction mediated by p53 class mediators (FDR: 0.0006) was also significantly downregulated. The top enriched genes in these downregulated processes were *Hsph1*, *Hsp90aa*, and *Hspa1b* (Fig 4c; S4 Fig). Western blot analysis further confirmed that the protein expression patterns of these representative genes were consistent with the RNA-seq results, with Vim, GPX1, Atf3, CD44, and Arpc5 showing higher expression in the B[a]P exposure group, whereas Hsph1, Hsp90aa, and Hspa1b showed lower expression in the B[a]P exposure group compared with the B[a]P withdrawal groups (Fig 4d).

### 3.4. Cell communication in premalignant lesions of mice during B[a]P exposure and after B[a]P withdrawal

**Fig 5:**
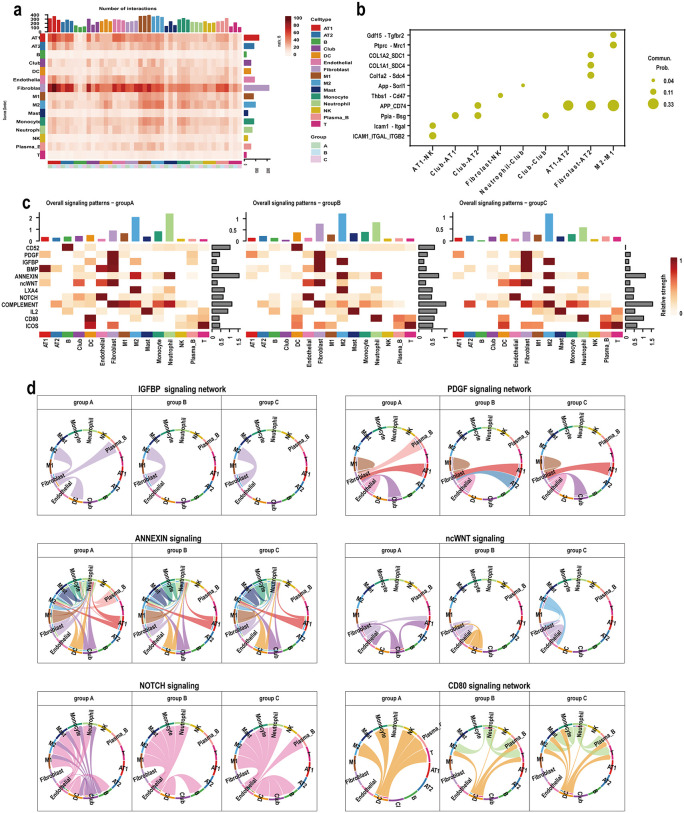
Comparative analysis of signaling pathways and intercellular communication in precancerous lung nodules between the B[a]P exposure group and graded B[a]P withdrawal groups. a, Heatmap showing the distribution of the number of intercellular communications mediated by signaling pathways among 15 cell types in groups A, B, and C. b, Bubble plot of key signaling pathways in intercellular communication between different cells. c, Heatmap showing the distribution of the number of intercellular communications mediated by individual signaling pathways among 15 cell types in groups A, B, and C. d, Circular plot of intercellular communication networks for six representative signaling pathways (IGFBP, PDGF, ANNEXIN, ncWNT, NOTCH, and CD80) in groups A, B, and C.

B[a]P withdrawal markedly remodelled the intercellular communication landscape of precancerous lung lesions. Compared with continued B[a]P exposure, B[a]P withdrawal reduced overall communication between AT1–NK, club–AT1, club–AT2, fibroblast–NK, neutrophil–club and club–club cell pairs (Fig 5a; S5 Fig). Pathway-level dissection identified Icam1–(Itgal+Itgb2) as the dominant mediator of AT1–NK signaling, Ppia–Bsg as the principal driver of club–AT1/AT2 and club–club interactions, and Thbs1–Cd47 as the major axis for fibroblast–NK communication; the activity of each of these pathways was significantly attenuated after B[a]P withdrawal. In contrast, APP–CD74 signaling was strengthened after B[a]P withdrawal, increasing information exchange between AT1–AT2, fibroblast–AT2 and M2–M1 cells (Fig 5b). To quantify pathway regulation we computed the information flow (defined as the sum of communication probabilities across all cell pairs) for each pathway: B[a]P withdrawal decreased information flow of CD52, PDGF, IGFBP, BMP, annexin, ncWNT, LAX4, complement, NOTCH and IL-2 pathways while upregulating CD80 and ICOS, with differences amplifying over longer cessation periods (S6 Fig). Single-pathway, cell-group heatmapping showed that IGFBP signaling shifted from fibroblasts in the B[a]P exposure state to M2 macrophages after cessation; BMP expression, previously contributed by fibroblasts and AT1 cells, became largely restricted to fibroblasts with a marked loss of AT1 contribution; ncWNT and NOTCH activities, formerly prominent in club and endothelial compartments, were substantially diminished in club cells following cessation (Fig 5c,d). Together, these results indicate that B[a]P exposure cessation alters cell composition and rewires specific signaling programs, implicating these pathway shifts in the regulatory effects of cessation on precancerous lesions.

### 3.5. Diversity of BCRs, TCRs, amino acid metabolism pathways, and gene regulation mechanisms in premalignant lesions of mice during B[a]P exposure and after B[a]P withdrawal

**Fig 6:**
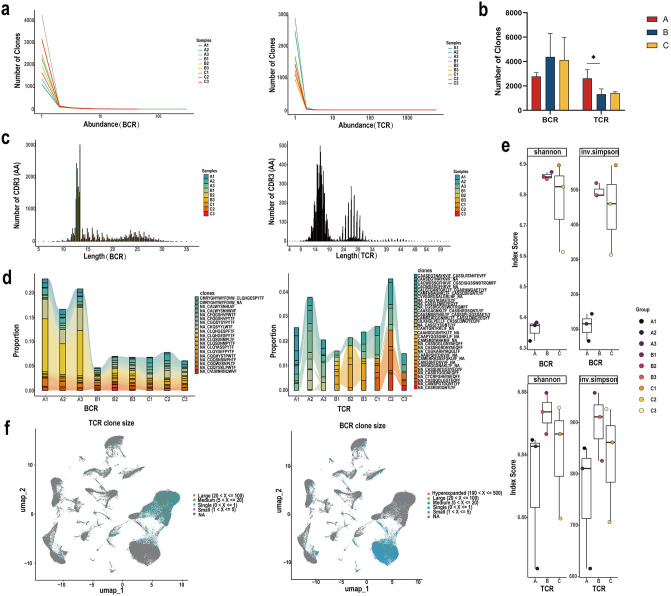
Clonal characteristics analysis of B- and T-cell receptor immune repertoires. a, Clonal abundance curves for B-cell receptors (BCRs) and T-cell receptors (TCRs). b, Statistical analysis of detectable sequence numbers for BCRs and TCRs. The bar chart shows the average clonality of BCRs and TCRs for each group, with error bars indicating standard deviation and * indicating P < 0.05. c, Amino acid sequence length distribution of complementary determining region 3 (CDR3) for BCRs and TCRs. d, Shared analysis of CDR3 amino acid sequences for BCRs and TCRs. The stacked bar chart displays the distribution of shared clones across groups with different colors representing different samples. e, Diversity analysis of BCRs and TCRs. The box plot compares the diversity indices across different groups. f, Distribution of BCR and TCR clones of varying sizes on the UMAP plot. UMAP dimensionality reduction shows the spatial distribution of clones of different sizes across samples with different colors representing clone size classifications.

IR-seq was used to identify 36,863 BCR sequences and 16,125 matching TCR sequences from lung precancerous lesions in the B[a]P exposure and B[a]P withdrawal groups. The CDR3 region clone abundance of BCRs and TCRs exhibited characteristic curves (Fig 6a), with few high-abundance clones and a distinct low-abundance tail effect. In the TCR low-abundance clone region, the number of CDR3 clones was significantly lower in the B[a]P withdrawal group than in the B[a]P exposure group (P < 0.05), whereas no significant difference in BCR CDR3 clone counts was observed between the two groups (Fig 6b). These results suggest that B[a]P withdrawal reduces the activity of the TCR CDR3 region. In the BCR CDR3 region, amplification was primarily concentrated in the 13-amino acid range, whereas for the TCR CDR3 region, amplification primarily occurred in the 13–18 and 22–31 amino acid ranges. Notably, in the 22–31 range, the number of amplified sequences was significantly higher in the B[a]P exposure group than in the B[a]P withdrawal group, suggesting that TCR sequences in this range may be pivotal for the regulatory effects of B[a]P withdrawal (Fig 6c). Shared sequence analysis of the CDR3 region revealed 19 common BCR CDR3 amino acid sequences, with NA_CLQHGESPYTF and NA_CLQHGESPFTF exhibiting group-specific differences. TCR CDR3 had one shared sequence (NA_CASSLDWGYEQYF); however, a unique sequence (NA_CASSRGRDNYAEQFF) was significantly amplified in the B[a]P withdrawal group. These findings suggest that NA_CLQHGESPYTF, NA_CLQHGESPFTF, and NA_CASSLDWGYEQYF may drive the malignant progression of B[a]P-induced precancerous lesions, whereas NA_CASSRGRDNYAEQFF may be a key sequence in the regulatory effects of B[a]P withdrawal (Fig 6d). Diversity analysis showed that both BCR and TCR diversity were lower in the B[a]P exposure group than in the post-exposure group (Fig 6e), suggesting that B[a]P exposure reduces BCR and TCR clonal diversity, which is restored upon cessation. UMAP visualization of BCR and TCR CDR3 region V, D, and J segment gene annotations combined with Seurat object clustering revealed that BCR small clones and monoclonal cells occupied most of the UMAP space, forming distinct clusters. In contrast, medium and large clones showed sparse distributions. TCR clones predominantly clustered in the NA region, with small and monoclonal cells occupying most of the space, whereas medium and large clones showed limited expansion and sparse distributions compared to BCR clones (Fig 6f). These results indicate substantial differences in immune cell regulation associated with B[a]P exposure and withdrawal, highlighting distinct mechanisms across these states.

### 3.6. Effects of B[a]P withdrawalon BCR and TCR amino acid usage frequency and VDJ gene expression

**Fig 7:**
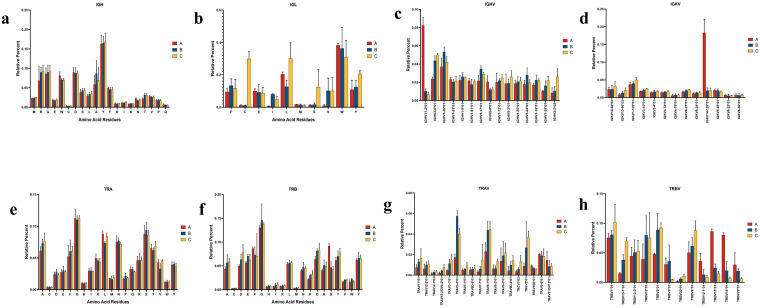
Amino acid and gene expression analysis of BCRs and TCRs. a, Amino acid expression differences of the IGH chain in BCRs. b, Differences in amino acid expression in the IGL chain in BCRs. c, Gene expression differences of the IGHV genes in BCRs. d, Gene expression differences of the IGKV genes in BCRs. e, Amino acid expression differences of the TRA chain in TCRs. f, Amino acid expression differences of the TRB chain in TCRs. g, Gene expression differences of the TCR α variable (TRAV) genes in TCRs. h, Gene expression differences of the TCR β variable (TRBV) genes in TCRs.

The amino acid usage frequency of the IGH and IGL chains of BCRs in the three groups was analyzed. For the IGH chain, tyrosine (Y), arginine (R), glycine (G), serine (S), and leucine (L) showed higher usage frequency in the B[a]P withdrawal group than in the B[a]P exposure group. In contrast, tryptophan (W), cysteine (C), aspartic acid (D), and valine (V) were more frequently used in the B[a]P exposure group than in theB[a]P withdrawal group (Fig 7a). In the IGL chain, S and Y were used more frequently in the B[a]P withdrawal group, whereas methionine (M) was more prevalent in the smoking group (Fig 7b). Notably, Y and S were significantly upregulated in both the IGH and IGL chains of the B[a]P withdrawal group, suggesting that Y, S, and M may play key roles in regulating precancerous lesions through B[a]P withdrawal.

Analysis of differential VDJ gene expression in the IGH chain revealed that IGHV1–39*01, IGHV2–2*02, IGHV5–17*01, and IGHV3–6*01 were significantly upregulated in the B[a]P withdrawal group, with their expression levels further increasing over time. IGHV3–6*01 exhibited the most significant differential expression. Conversely, IGHV11–2*01 was significantly downregulated in the B[a]P withdrawal group (Fig 7c). For the IGL chain, IGKV10–94*03, IGKV10–96*03, IGKV1–135*01, IGKV3–10*01, IGKV3–4*01, IGKV4–74*01, IGKV4–78*01, IGKV8–21*01, and IGKV8–24*01 were upregulated, with expression levels increasing over time. In contrast, IGKV14–126*01, IGKV5–45*01, IGKV6–25*01, IGKV8–30*01, and IGLV2*02 were downregulated (Fig 7d).

Amino acid usage frequency analysis of the T-cell receptor alpha (TRA) and T-cell receptor beta (TRB) chains in TCRs revealed distinct patterns. In the TRA chain, alanine (A) was used more frequently in the B[a]P withdrawal group, whereas histidine (H), isoleucine (I), and lysine (K) showed higher usage in the B[a]P exposure group (Fig 7e). For the TRB chain, A, C, D, K, L, M, proline, glutamine, and T were more frequently used in the B[a]P withdrawal group than in the B[a]P exposure group, with their frequencies increasing over time, whereas phenylalanine and S showed significant decreases (Fig 7f). The consistent upregulation of A in both the TRA and TRB chains suggests its crucial role in activating immature and memory T-cells. The high expression of A after B[a]P withdrawal indicates enhanced T-cell maturation and immune activity, with both increasing over time.

Differential expression analysis of TRA chain VDJ genes showed that TRAV15–2/DV6–2*01, TRAV3−1*01, TRAV8−1*01, TRAV8−2*01, TRGV1*02, TRAV7−3*01, TRAV3−3*01, and TRAV12D-1*02 were upregulated in the B[a]P withdrawal group, with TRAV3−3*01 exhibiting the most significant differential expression among the three groups. However, TRAV10D*01 was significantly downregulated (Fig 7g). In the TRB chain, TRBV1*01, TRBV13−1*01, TRBV13−3*01, TRBV19*01, TRBV2*01, TRBV3*01, TRBV30*01, and TRBV4*01, were upregulated, whereas TRBV12−1*01, TRBV15*01, TRBV16*01, and TRBV20*01 were downregulated (Fig 7h). The detailed list and functional annotations of the differentially expressed amino acids and genes are provided in S2 Table.

## 4. Discussion

Chronic exposure to tobacco carcinogens disrupts alveolar epithelial homeostasis, particularly in AT2 cells, and promotes epithelial–mesenchymal transition (EMT) and cancer stem cell (CSC) formation during lung preneoplastic progression [1]. However, whether these changes are reversible after smoking cessation remains unclear. Here, using B[a]P withdrawal as an experimental surrogate for smoking cessation, we combined scRNA-seq and IR-seq to examine lung preneoplastic lesions and observed coordinated alterations in epithelial, immune, and molecular features.

At the signaling level, B[a]P withdrawal suppressed multiple pro-tumorigenic pathways, including Icam1-(Itgal+Itgb2), Ppia–Bsg, and Thbs1–Cd47, while enhancing the APP–CD74 axis. The downregulation of Icam1-(Itgal+Itgb2), Ppia–Bsg, and Thbs1–Cd47 is consistent with reduced inflammatory signaling, epithelial plasticity, and early carcinogenic changes. The Icam1-(Itgal+Itgb2) pathway is known to participate in leukocyte adhesion and inflammatory responses [22], and its suppression may reflect reduced inflammatory cell recruitment and tissue injury. Suppression of the Ppia–Bsg pathway, which has been associated with tumor cell proliferation, migration, drug resistance, and CSC-like properties [23], together with inhibition of the Thbs1–Cd47 pathway, which has been implicated in the regulation of γ-catenin and E-cadherin interactions during EMT [24], is consistent with reduced stem-like features and partial attenuation of mesenchymal transition after B[a]P withdrawal. In contrast, enhanced APP–CD74 signaling may indicate a role in immune modulation, inflammation resolution, and restoration of microenvironmental homeostasis [25]. Notably, B[a]P withdrawal also reshaped intercellular communication, including that between AT1 and AT2 cells, fibroblasts and AT2 cells, and M1 and M2 macrophages, further supporting a potential contribution of the APP–CD74 axis to tissue repair and immune remodeling in lung preneoplastic lesions.

Additionally, B[a]P withdrawal was associated with the downregulation of the CD52, PDGF, IGFBP, BMP, annexin, ncWNT, LAX4, and NOTCH signaling pathways, together with the upregulation of the CD80 and ICOS pathways. CD52, predominantly expressed on lymphocytes, has been implicated in non–small cell lung cancer proliferation and migration via the AKT signaling pathway; thus, its reduced signaling after withdrawal may help weaken this pro-tumorigenic route [26]. The concurrent reduction of PDGF- and BMP-related signaling is also consistent with attenuation of pro-tumor processes related to epithelial plasticity, stromal and microenvironmental remodeling, and immune dysregulation [27–30]. NOTCH signaling, which has been implicated in lung adenocarcinoma progression and in cancer-associated fibroblast-mediated metastatic activity, was likewise suppressed [31,32]. Concurrently, the upregulation of CD80 and ICOS signaling is consistent with enhanced immune co-stimulation after withdrawal [33,34]. Together, these changes indicate that B[a]P withdrawal not only dampens several pro-tumor signaling networks but also reinforces adaptive immune activity, thereby supporting a plausible biological basis for the more favorable resolution of preneoplastic lesions.

At the transcriptional level, B[a]P withdrawal reduced the expression of epithelial–mesenchymal transition (EMT)- and cancer stem cell (CSC)-associated genes, including Vim, CD44, and Arpc5 [17–20], while also modulating stress response- and ferroptosis-related regulators, including GPX1 and Atf3 [21,35]. These findings suggest that B[a]P exposure is associated with molecular features of preneoplastic progression, characterized by increased epithelial plasticity and reduced ferroptosis, whereas B[a]P withdrawal partially reverses these changes. In addition, heat shock protein (HSP) family members, including Hsph1, Hsp90aa, and Hspa1b, were upregulated following B[a]P withdrawal [36], indicating an enhanced cellular stress response. Although their roles in lung preneoplastic lesions remain incompletely defined, this response may help maintain proteostasis and limit further lesion progression. These transcriptional changes were further supported at the protein level by western blot analysis, which showed higher expression of Vim, GPX1, Atf3, CD44, and Arpc5 in the B[a]P exposure group, whereas Hsph1, Hsp90aa, and Hspa1b showed lower expression than in the B[a]P withdrawal groups.

Immune repertoire analysis further revealed dynamic remodeling of adaptive immunity. Analysis of BCR and TCR clonal abundance, diversity, and CDR3 amino acid sequence distribution showed that B[a]P exposure reduced repertoire diversity and promoted clonal expansion, whereas B[a]P withdrawal was associated with gradual restoration of diversity, suggesting improved immune adaptability. In particular, TCR CDR3 sequences in the B[a]P exposure group showed increased expansion within specific length ranges, consistent with selective clonal activation under smoking-related conditions. Together, these findings suggest that B[a]P withdrawal, used here as an experimental proxy for smoking cessation, may contribute to partial restoration of immune homeostasis and immune surveillance in preneoplastic lesions, consistent with recent evidence that some smoking-related immune alterations are reversible after cessation, whereas others may persist [37]. Notably, distinct TCR clonotypes were observed between B[a]P exposure and withdrawal conditions, with enrichment of NA_CASSLDWGYEQYF in the B[a]P exposure group and NA_CASSRGRDNYAEQFF following withdrawal, indicating divergent immune states associated with disease progression or resolution and highlighting their potential relevance as markers of immune remodeling, although this will require further validation.

In addition, B[a]P withdrawal altered amino acid usage patterns in immune receptors, including increased utilization of methionine, tyrosine, leucine, and alanine. Methionine contributes to methyl donor supply through S-adenosylmethionine and has been implicated in tumor-associated metabolic dependencies [38], whereas restriction of methionine metabolism or extracellular methionine availability has been reported to suppress tumor growth and impair tumor cell survival [39]. Tyrosine showed an upward trend in both the IGH and IGL chains of BCRs and may be associated with altered antibody-binding properties, given the prominent role of tyrosine residues in antibody-antigen interfaces [40]. Leucine transport through amino acid transporters such as SLC7A5 has been linked to mTORC1 activation in lymphocytes and to regulation of T-cell differentiation and cytokine production [41]. Alanine usage was increased in both TRA and TRB chains of TCRs and may be related to naïve T-cell activation and memory T-cell restimulation, thereby contributing to antigen-specific immune responses [42]. In addition, this study systematically assessed the impact of B[a]P withdrawal on BCR and TCR V(D)J usage. In the IGH repertoire, cessation markedly increased IGHV3–601 expression while decreasing IGHV11–201, suggesting remodeling of antigen receptor usage patterns. Similarly, in the TCR repertoires, upregulation of TRAV3–301 and TRBV13–101 after cessation further supports adaptive immune receptor remodeling under B[a]P exposure and withdrawal conditions [43,44]. However, the functional significance of these repertoire alterations remains to be determined.

Overall, these findings suggest that B[a]P withdrawal, used here to model smoking cessation, is associated with attenuation of molecular features linked to lung preneoplastic progression through coordinated regulation of epithelial plasticity, immune signaling, and repertoire diversity, highlighting a systems-level response that may contribute to lesion stabilization and potential regression.

## 5. Conclusions

In this B[a]P-based model, withdrawal of B[a]P exposure, used here to mimic smoking cessation, was associated with reduced lung preneoplastic nodule burden and restricted lesion growth in mice, along with reduced cellular atypia and alterations in immune cell composition, including increased T, B, and NK cells and decreased monocytes and neutrophils. These findings are consistent with smoking cessation-like effects on both epithelial and immune features of lung preneoplastic lesions.

B[a]P withdrawal was also associated with modulation of key molecular and immune pathways, including upregulation of CD80 and ICOS signaling, as well as remodeling of adaptive immune receptor repertoires, indicating altered immune adaptability within the preneoplastic microenvironment. Together, these changes suggest that smoking cessation-like intervention may help restrain preneoplastic progression through coordinated regulation of the cellular microenvironment and gene expression changes.

However, several limitations should be considered when interpreting these findings. The B[a]P-induced model does not fully recapitulate the complexity of cigarette smoke exposure. In the scRNA-seq and IR-seq analyses, comparisons were primarily conducted between the model and B[a]P withdrawal groups without simultaneous control–model comparisons, which may introduce confounding factors such as time-dependent changes. Therefore, causal interpretations should be made with caution. Further clinical and mechanistic studies are required to validate these findings.

Taken together, these results provide a framework for understanding the molecular and immunological changes associated with B[a]P withdrawal in lung preneoplastic lesions and support further investigation into the regulatory effects of smoking cessation on early lung carcinogenesis.

## Supporting information

S1 Fig UMAP plot of single-cell RNA-seq data from B[a]P-induced lung preneoplastic lesion tissue. The axes represent the two-dimensional coordinates generated by Uniform Manifold Approximation and Projection (UMAP). Different colors indicate different cell clusters, and the legend shows the cluster identities.
(TIF)

S2 Fig Dot plot of marker gene expression across cell subgroups. The x-axis shows the differentially expressed genes (DEGs) identified in each cell subgroup, and the y-axis shows the cell subgroups. Dot color represents the average expression level of each gene in the corresponding subgroup, and dot size indicates the proportion of cells expressing that gene.
(TIF)

S3 Fig Heatmap of Gene Ontology biological process enrichment in the B[a]P exposure and B[a]P withdrawal groups. Darker colors indicate greater enrichment fold change and stronger upregulation.
(TIF)

S4 Fig Heatmap of differentially expressed gene enrichment between the B[a]P exposure and B[a]P withdrawal groups. Darker colors indicate greater enrichment fold change and stronger upregulation.
(TIF)

S5 Fig Differential cell–cell communication network between the B[a]P exposure and B[a]P withdrawal groups. The size of each colored circle on the periphery represents the number of cells, with larger circles indicating more cells. Arrows indicate ligand-to-receptor interactions, and the arrowheads point to receptor-expressing cells. Thicker lines indicate a greater number of ligand–receptor pairs. Line color indicates changes in intercellular communication between the two groups, with blue representing decreased communication and red representing increased communication.
(TIF)

S6 Fig Signaling pathway flowcharts comparing group A versus group B, group B versus group C, and group A versus group C. (TIF)

S1 Table Differentially expressed genes in the B[a]P exposure group versus the B[a]P withdrawal group and Gene Ontology biological process enrichment results. (DOCX)

S2 Table Amino acid types, differentially expressed genes upregulated or downregulated after B[a]P withdrawal, and their functional annotations. (DOCX)

S1 Raw images Original uncropped and unadjusted western blot images corresponding to Fig 4d. (PDF)
