# Supplementary material for: Regulatory effects of smoking cessation on the cellular microenvironment and differentially expressed genes in precancerous lesions of pulmonary nodules in mice based on single-cell RNA sequencing and immune repertoire-sequencing
Source: PLoS One. 2026 Aug 13;21(8):e0356148. doi: 10.1371/journal.pone.0356148 (PMC13472401; doi:10.1371/journal.pone.0356148)

S1 Raw images

Original uncropped and unadjusted western blot images for Fig 4d

Fig 4d. Western blot validation of representative proteins.
β-Actin was used as the shared loading control for all target proteins.

Lane order:
M: molecular weight marker
Lane 1: Group A, B[a]P exposure
Lane 2: Group B, B[a]P withdrawal for 4 weeks
Lane 3: Group C, B[a]P withdrawal for 8 weeks
Lane 4: Group A, B[a]P exposure
Lane 5: Group B, B[a]P withdrawal for 4 weeks
Lane 6: Group C, B[a]P withdrawal for 8 weeks
Lane 7: Group A, B[a]P exposure
Lane 8: Group B, B[a]P withdrawal for 4 weeks
Lane 9: Group C, B[a]P withdrawal for 8 weeks
The red box indicates the region cropped for presentation in Fig 4d. Lanes marked with "X" were not included in the final representative western blot panel.

Fig 4d - Vimentin / Vim

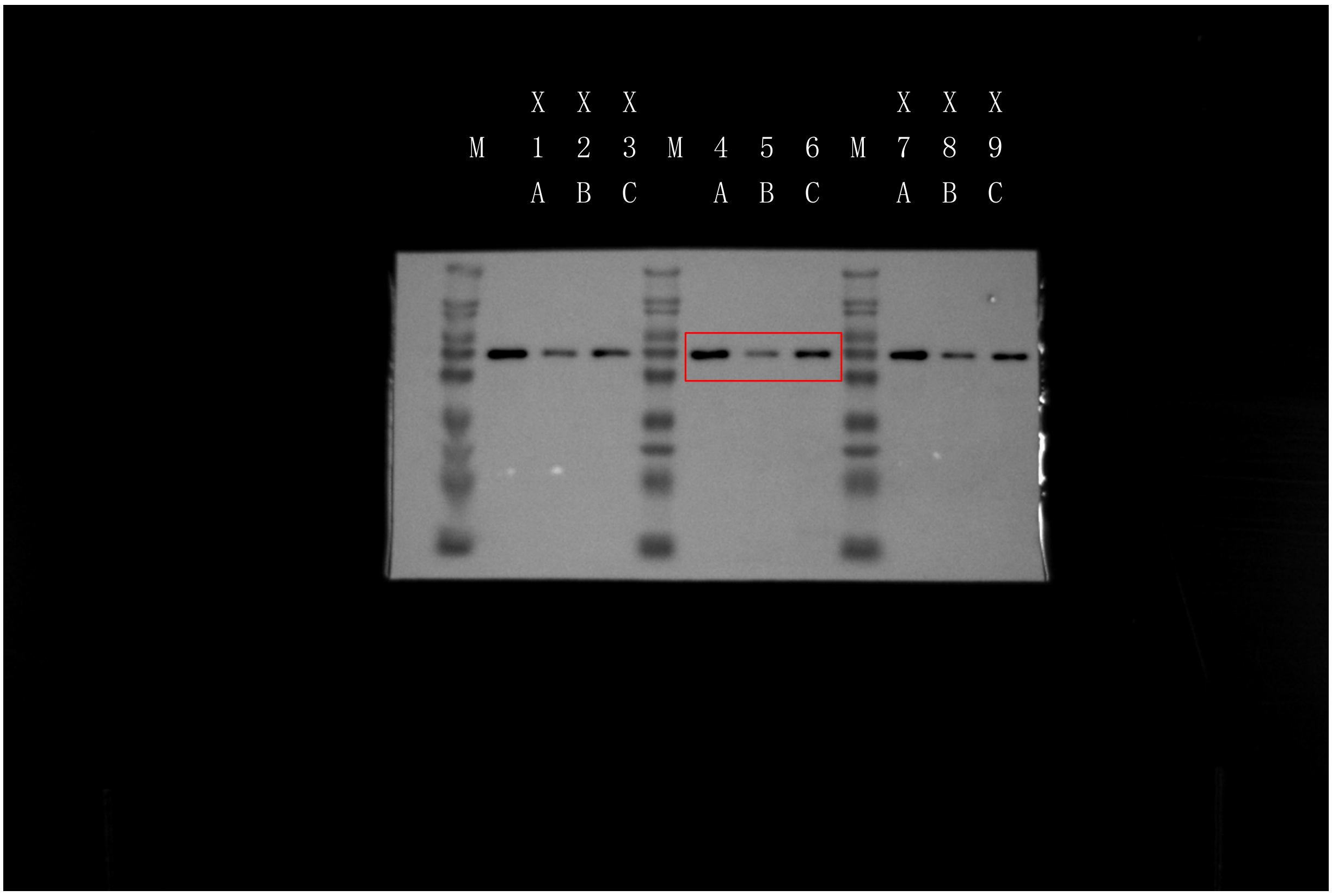

Fig 4d. Original uncropped and unadjusted western blot images
Lane order is the same as Page 1.

Fig 4d - GPX1

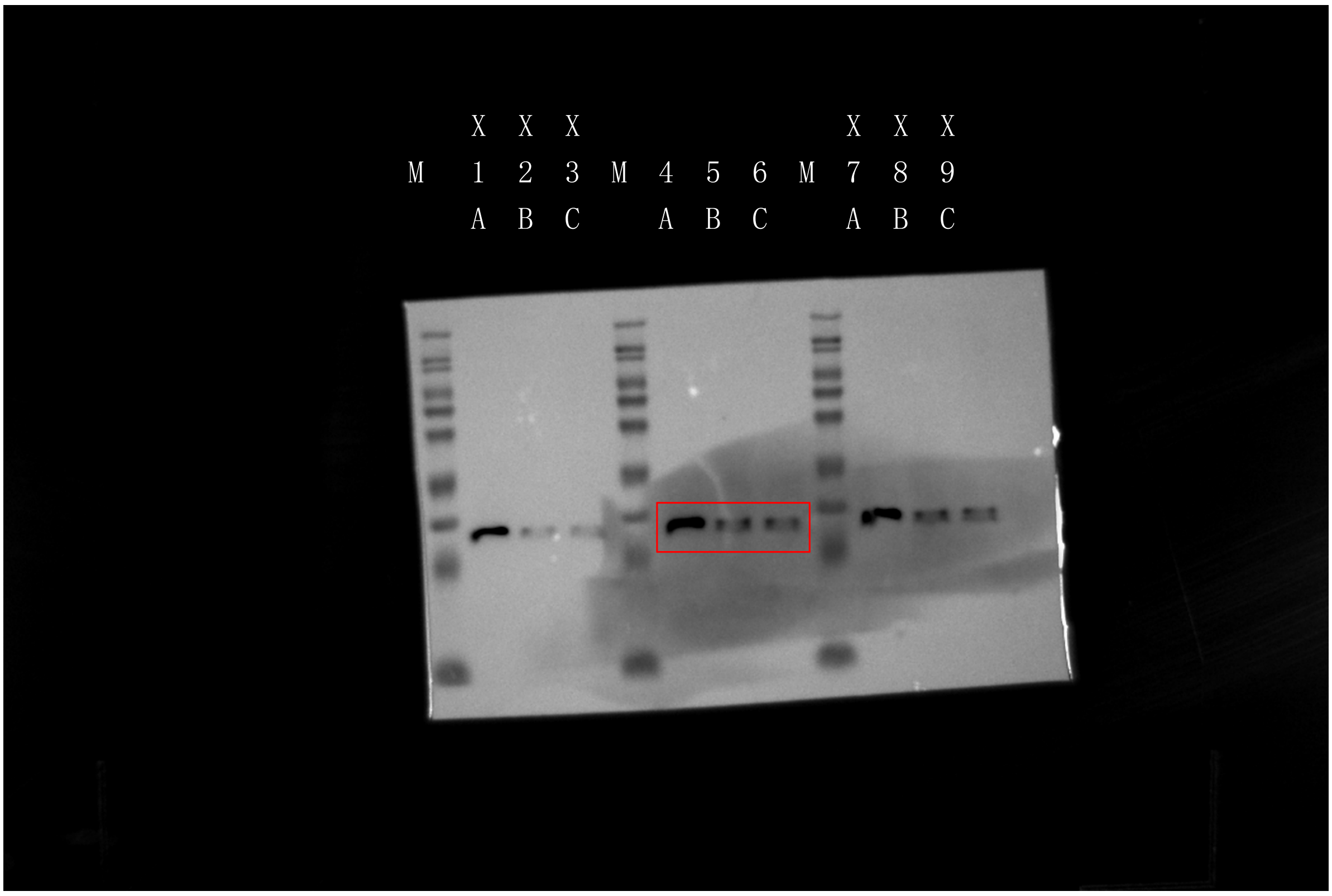


Fig 4d - ATF3

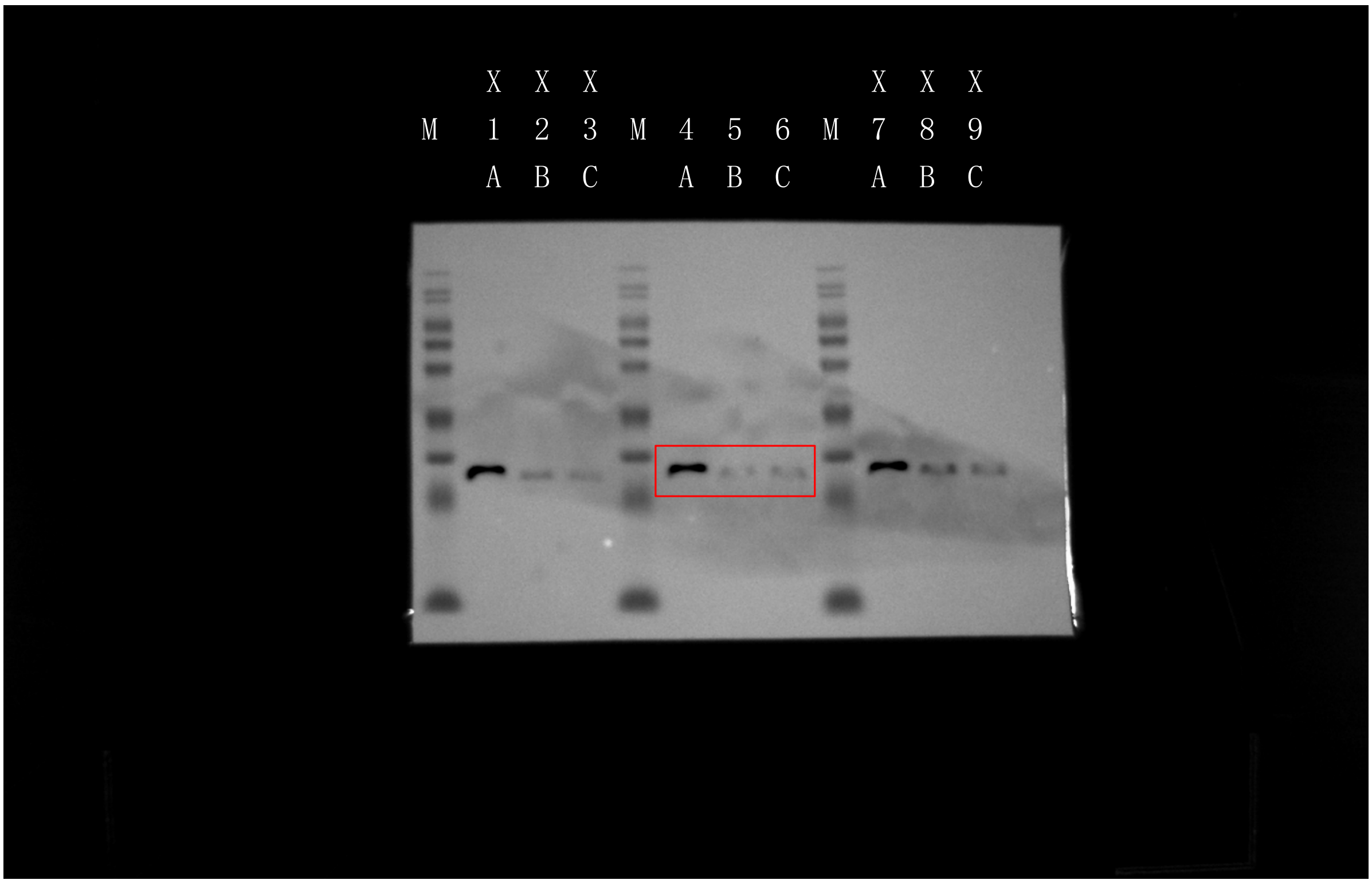

Fig 4d. Original uncropped and unadjusted western blot images
Lane order is the same as Page 1.

Fig 4d - CD44

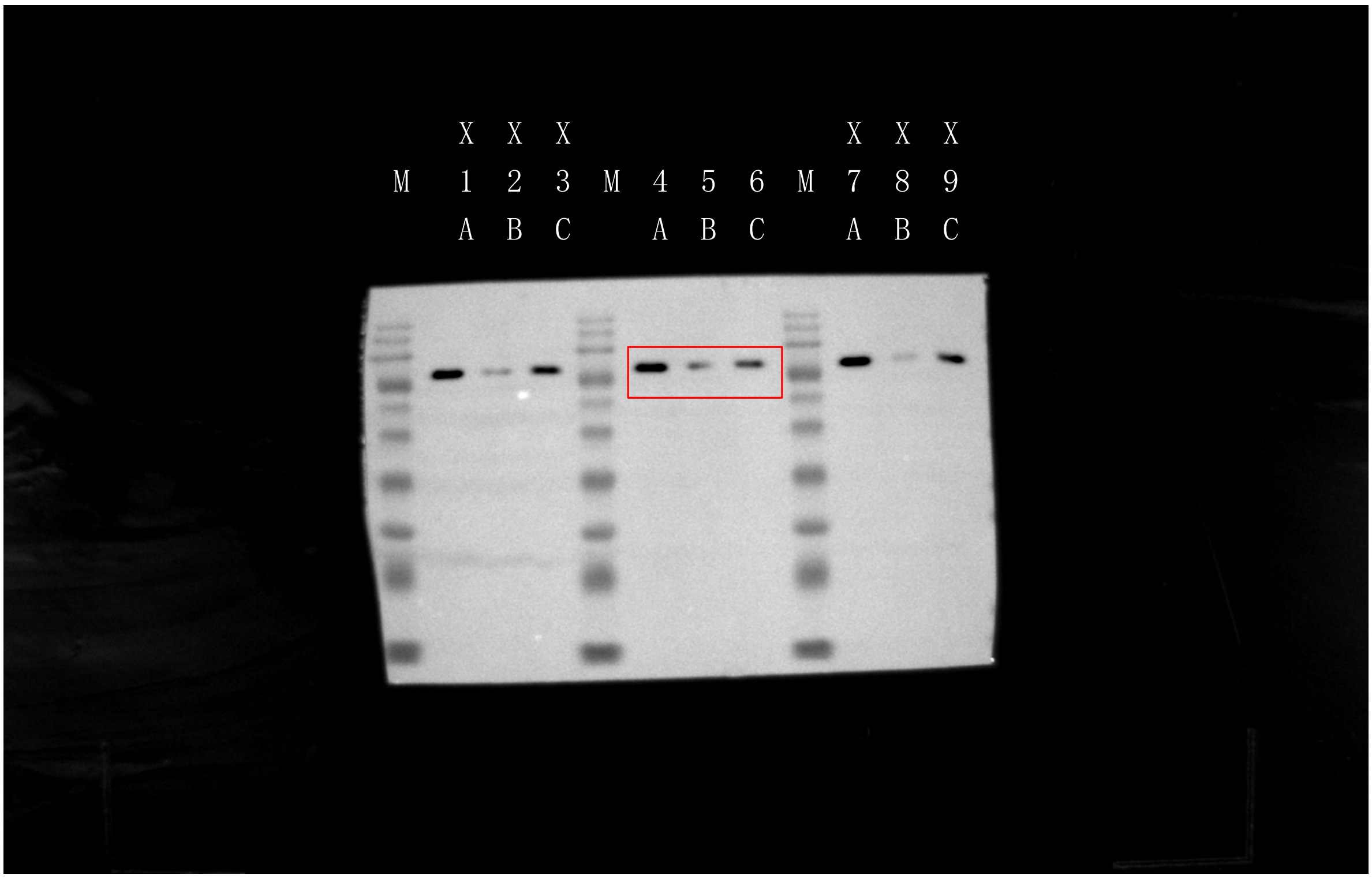


Fig 4d - ARPC5

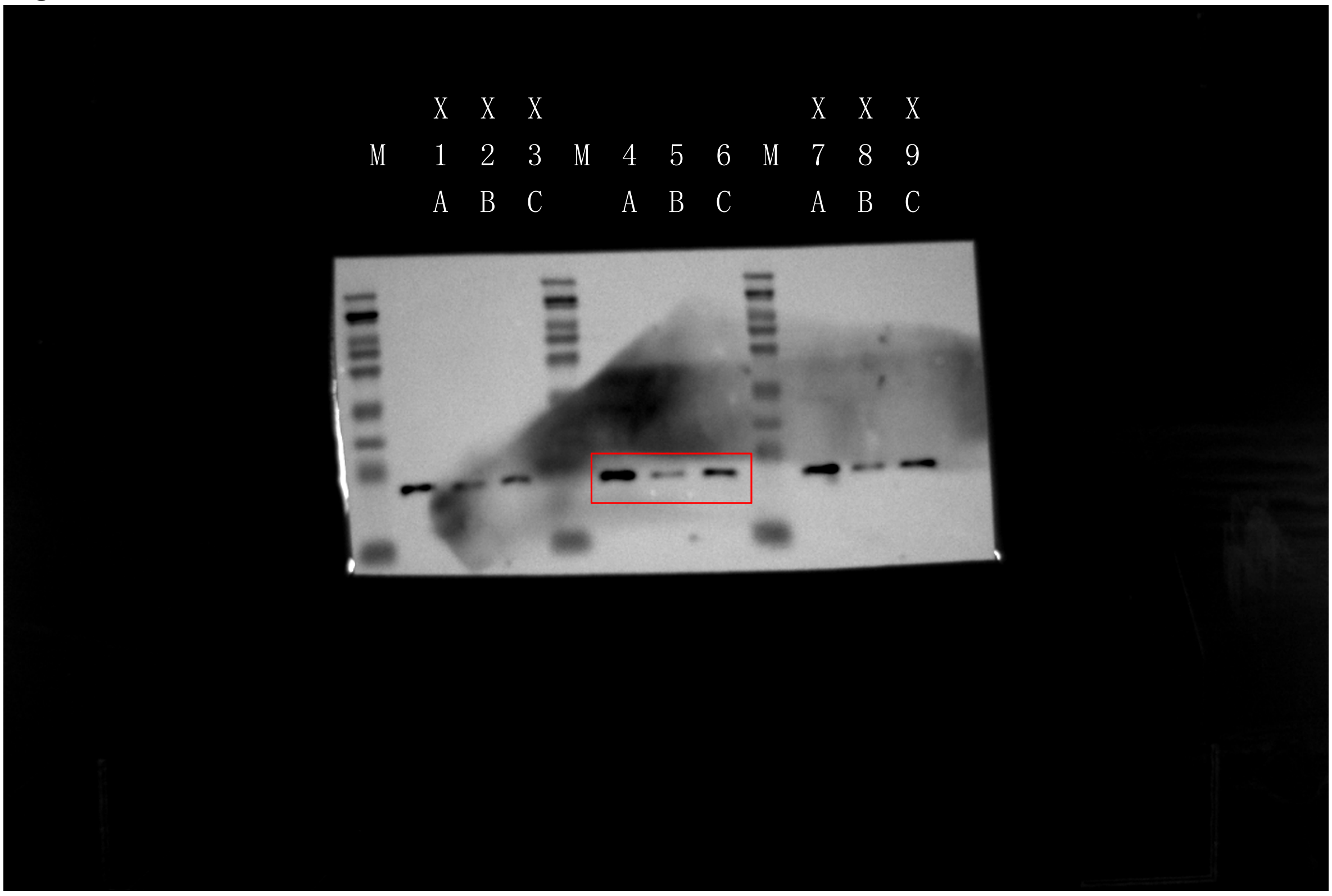

Fig 4d. Original uncropped and unadjusted western blot images
Lane order is the same as Page 1.

Fig 4d - Hsp105 / Hsph1

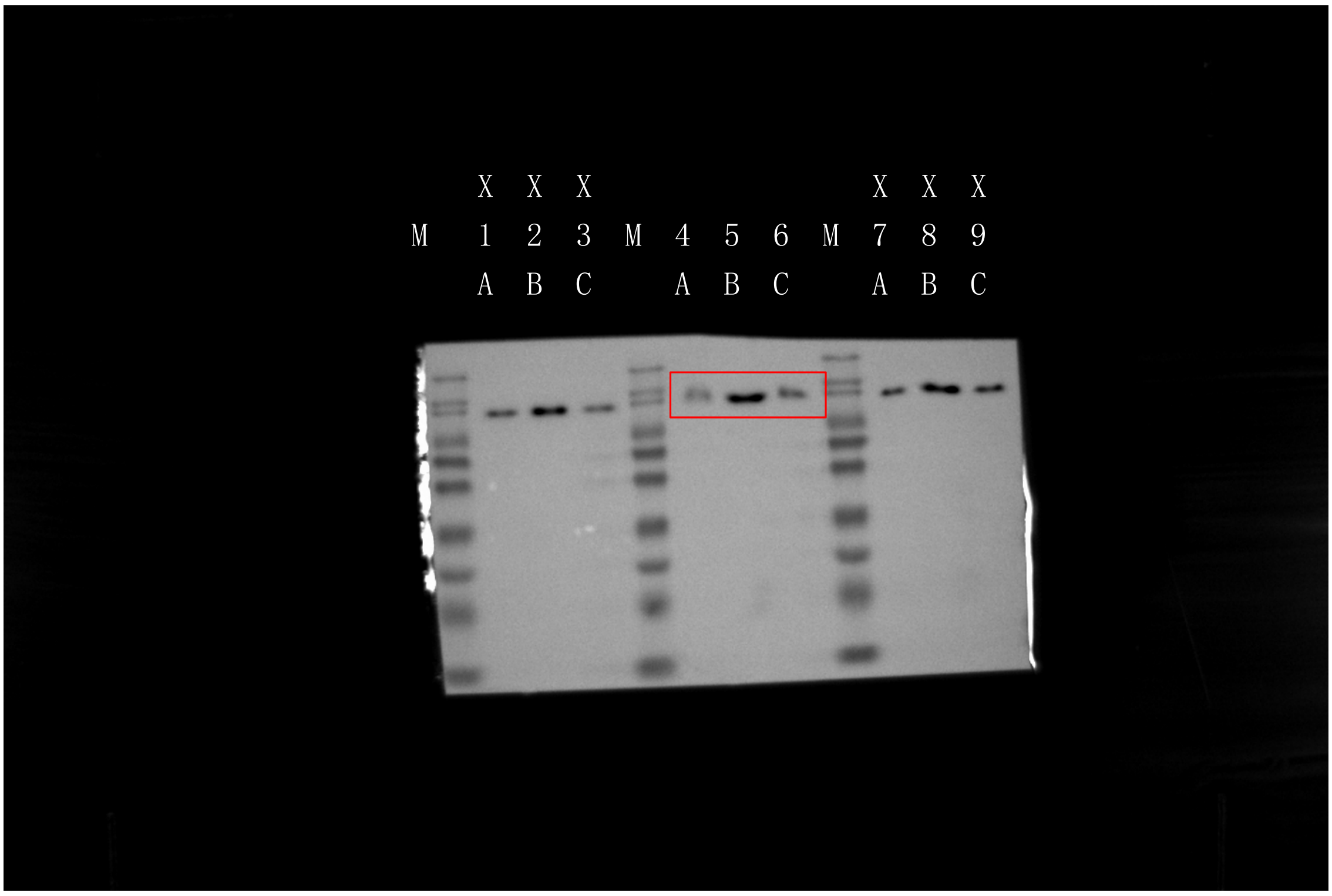


Fig 4d - Hsp90 / Hsp90aa

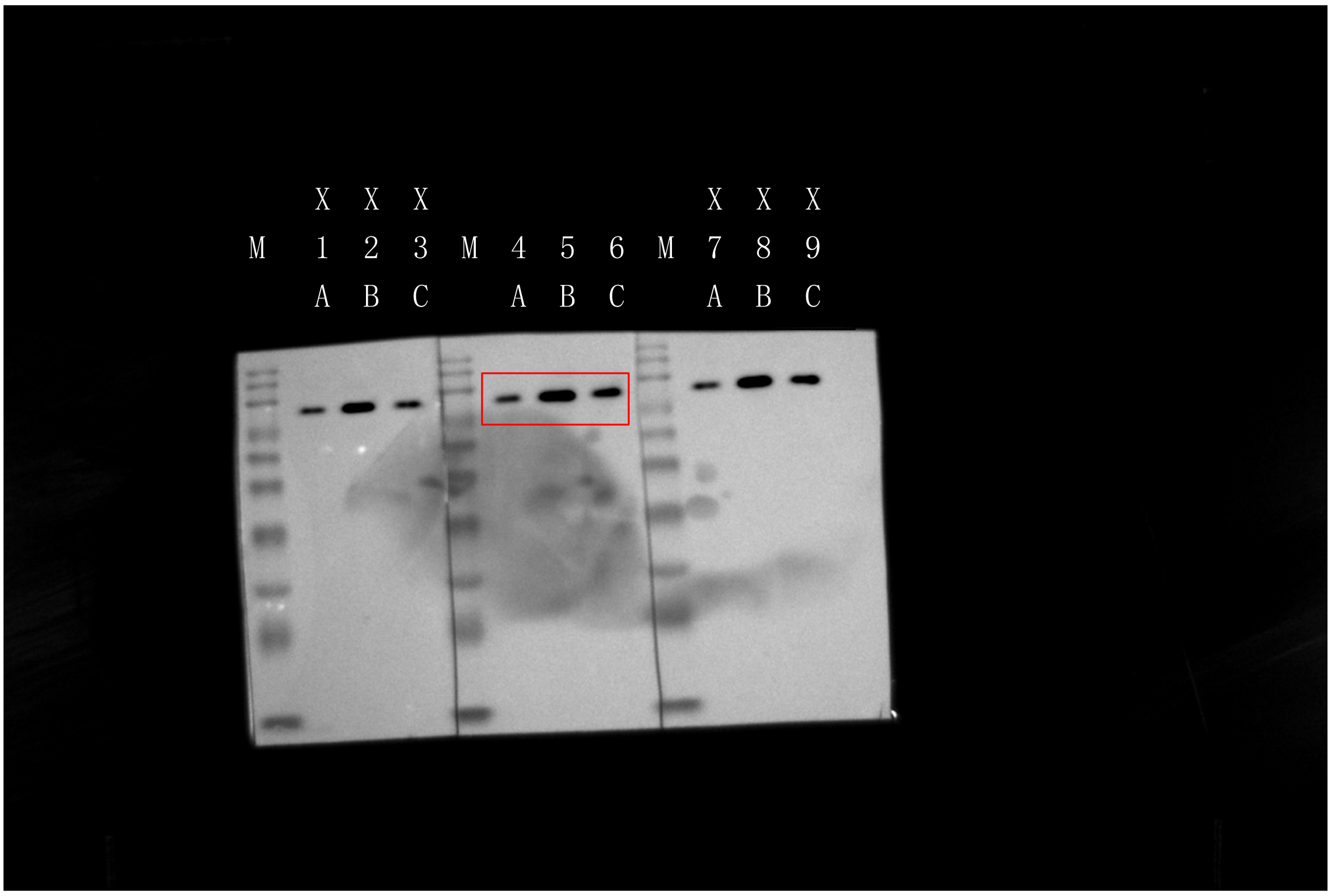

Fig 4d. Original uncropped and unadjusted western blot images
Lane order is the same as Page 1.

Fig 4d - Hsp70 / Hspa1b

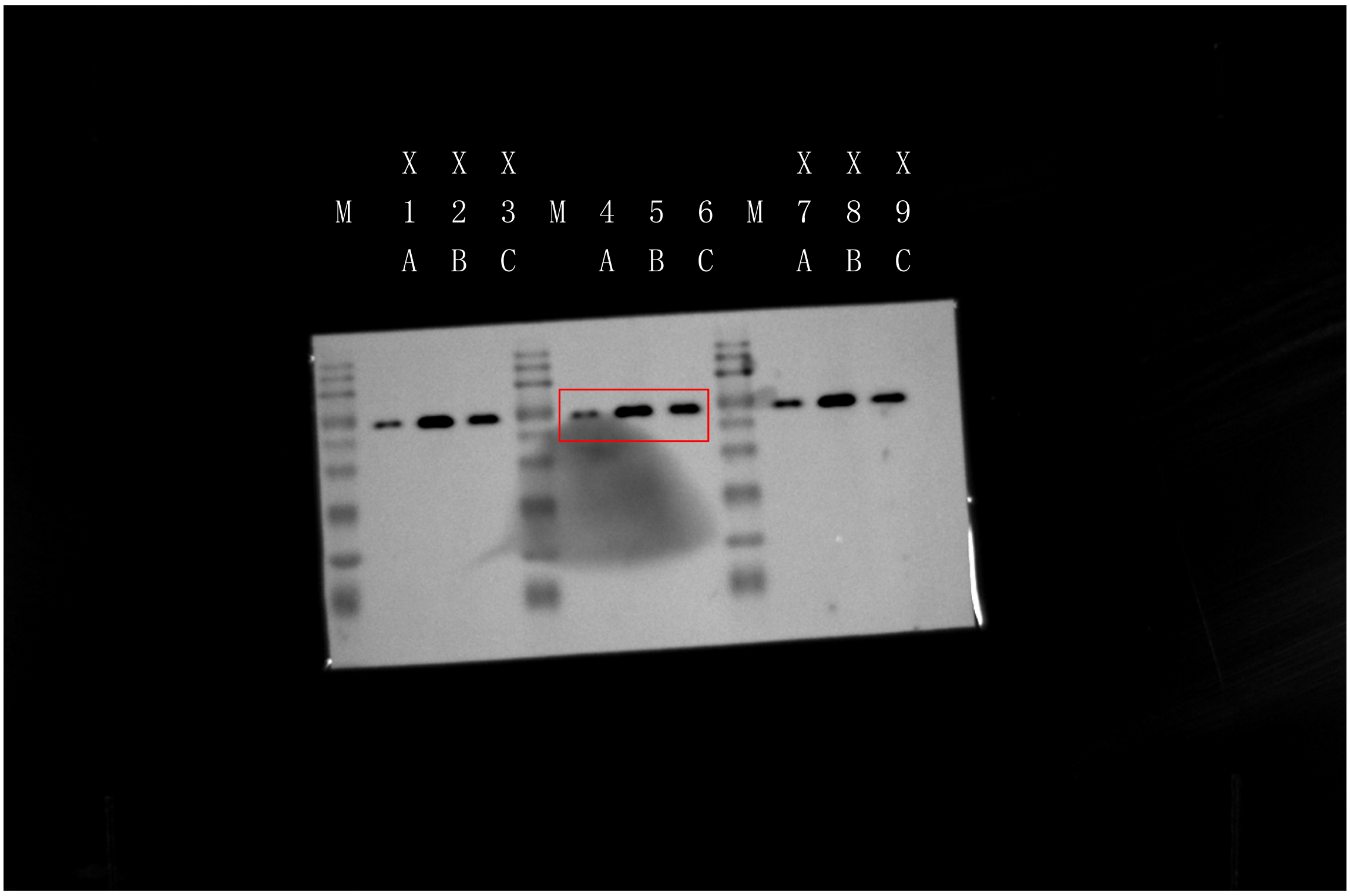

Fig 4d. Original uncropped and unadjusted western blot image for shared β-Actin loading control

β-Actin was used as the shared loading control for Vimentin/Vim, GPX1, ATF3, CD44, ARPC5, Hsp105/Hsph1, Hsp90/Hsp90aa, and Hsp70/Hspa1b.

Lane order is the same as Page 1.

Fig 4d - β-Actin

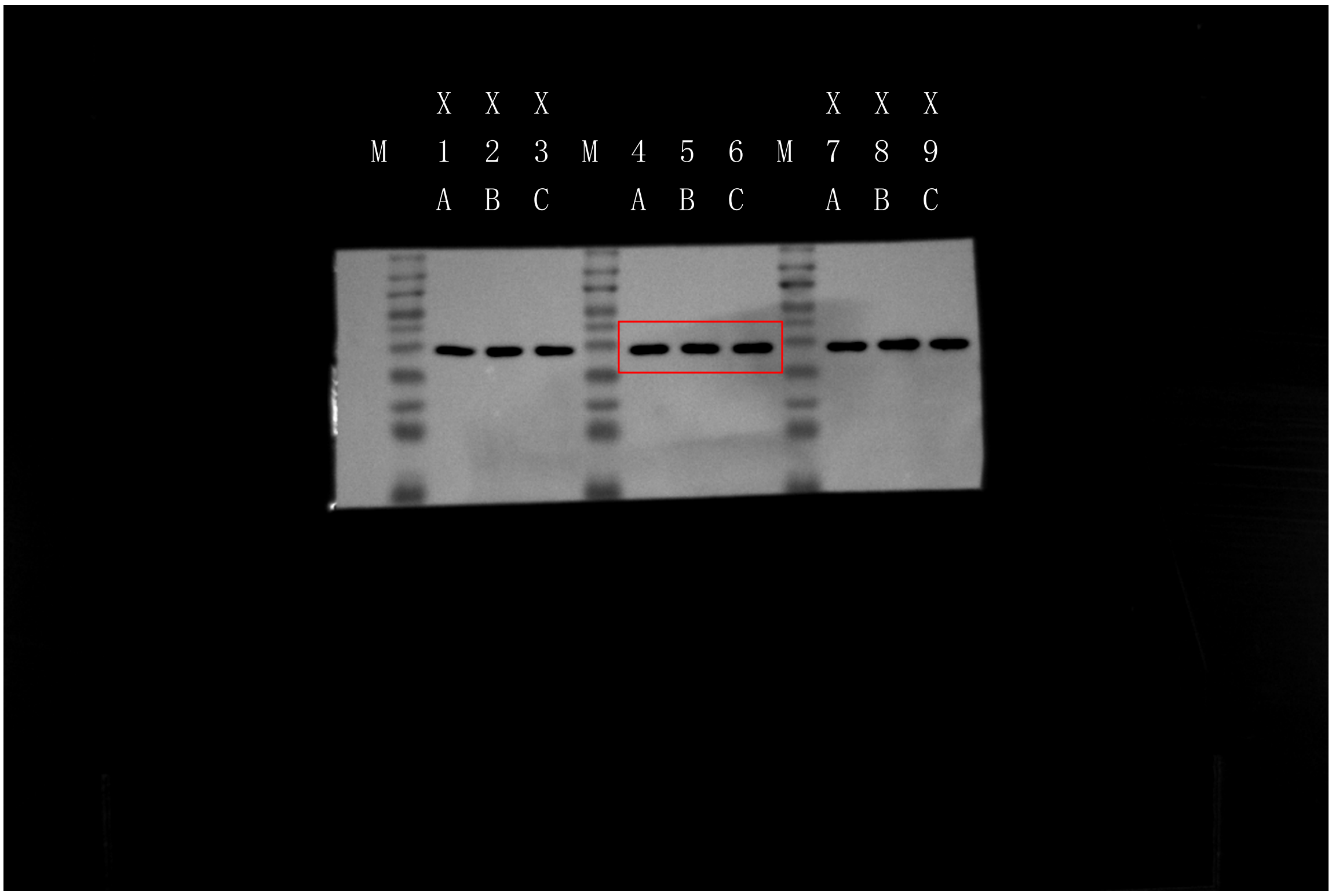

Supplement: Supplementary file 1 [file pone.0356148.s009.pdf]
