# Supplementary material for: Regulatory effects of smoking cessation on the cellular microenvironment and differentially expressed genes in precancerous lesions of pulmonary nodules in mice based on single-cell RNA sequencing and immune repertoire-sequencing
Source: PLoS One. 2026 Aug 13;21(8):e0356148. doi: 10.1371/journal.pone.0356148 (PMC13472401; doi:10.1371/journal.pone.0356148)
Supplement: Supplementary file 2 [file pone.0356148.s007.docx]

S1 Table

Differentially expressed genes in the B[a]P exposure group versus the B[a]P withdrawal group and Gene Ontology biological process enrichment results.

| GO ID | GO Term | Adjusted p-value | Genes | Enrichment Fold |
| --- | --- | --- | --- | --- |
| GO:0032970 | Regulation of actin filament-based process | 1.76e-10 | Rac1/Sri/Arpc2/Arpc5/Capza2/Plek/Prkcd/Actr3/Capzb/Arpc1b/ Mtpn/  Vasp/Iqgap1/Flna/Tmsb4x/Cfl1/Actg1/Rhog | 10.26 |
| GO:0043254 | Regulation of protein complex assembly | 8.16e-10 | Rac1/Arpc2/Arpc5/Capza2/Sgk1/Plek/Prkcd/Lcp1/Actr3/Lmo4/ Capzb/  Arpc1b/Mtpn/Vasp/Sh3glb1/Tmsb4x/Cfl1 | 9.51 |
| GO:1902903 | Regulation of supramolecular fiber organization | 1.97e-09 | Rac1/Arpc2/Arpc5/Capza2/Sgk1/Plek/Prkcd/Actr3/Capzb/Arpc1b/Mtpn/Vasp/Flna/Tmsb4x/Cfl1/Actg1 | 9.75 |
| GO:0051258 | Protein polymerization | 2.37e-09 | Rac1/Arpc2/Arpc5/Capza2/Ndel1/Sgk1/Prkcd/Actr3/Capzb/Arpc1b/Mtpn/Vasp/Tmsb4x/Cfl1 | 12.10 |
| GO:0032535 | Regulation of cell size | 2.66e-08 | Rac1/Arpc2/Arpc5/Capza2/Ndel1/Plek/Prkcd/Actr3/Capzb/Arpc1b/Mtpn/  Vasp/Picalm/Tmsb4x/Cfl1 | 8.96 |
| GO:1901653 | Response of cells to peptide | 1.94e-06 | Rac1/Ctnnb1/Nfe2l2/Ndel1/Sgk1/Prkcd/Adipor1/Vim/Pkm/Gnai2/ Socs3/Cfl1 | 8.86 |
| GO:2001233 | Regulation of apoptotic signaling pathway | 2.18e-06 | Cd44/Ctnnb1/Nfe2l2/Prelid1/Prkcd/Tmbim6/Atf3/Pdia3/Ctsc/Gnai2//Plaur/Gpx1 | 7.69 |
| GO:1901652 | Response to peptide | 2.49e-05 | Rac1/Ctnnb1/Nfe2l2/Ndel1/Sgk1/Prkcd/Adipor1/Vim/Pkm/Gnai2/Socs3/Cfl1 | 6.65 |
| GO:0006801 | Superoxide metabolic process | 3.76e-05 | Itgb2/Cybb/Nfe2l2/Prkcd/Prdx1/Gnai2 | 20.98 |
| GO:0042060 | Wound healing | 4.65e-05 | Cd44/Nfe2l2/Plek/Prkcd/Itgb1/Flna/Lyn/Plaur/Actg1/Gpx1/Ahnak | 6.88 |
| GO:0002181 | Cytoplasmic translation | 8.59e-44 | Rpl8/Rplp1/Rps18/Rps15a/Rps5/Rpl19/Rps24/Rplp2/Rps20/Rps3/Rpsa/  Rps29/Rps16/Rpl10a/Rpl12/Rps21/Rpl22l1/Rps19/ Rpl18a/Rps8/ Rps23  /Rpl32/Rpl36/Rpl30/Rpl18/Rpl3/Rpl35a/Rps7/Rps17/Rps12/Rps15/Rplp0 | 52.13 |
| GO:0042274 | Ribosomal small subunit biogenesis | 1.39e-11 | Rps5/Rps24/Rpsa/Rps16/Rps21/Rps19/Rps8/Rps7/Rps17/Rps15/Rps27 | 35.59 |
| GO:0006364 | rRNA processing | 9.77e-07 | Rps24/Rps16/Rpl10a/Rps21/Rps19/Rps8/Rpl35a/Rps7/Rps17/Rps15/Rps27 | 11.92 |
| GO:0016072 | rRNA metabolic process | 4.63e-06 | Rps24/Rps16/Rpl10a/Rps21/Rps19/Rps8/Rpl35a/Rps7/Rps17/Rps15/Rps27 | 10.09 |
| GO:0042026 | Protein refolding | 1.81e-05 | Hspa8/Hsp90aa1/Dnaja1/Hspa1b/Hspa1a | 46.59 |
| GO:0042255 | Ribosome assembly | 3.53e-05 | Rps5/Rpsa/Rps19/Rps15/Rplp0/Rps27 | 23.29 |
| GO:0051085 | Co-chaperone-dependent protein refolding | 4.20e-05 | Dnajb1/Hspa8/Hsph1/Hspa1b/Hspa1a | 35.30 |
| GO:0071826 | Organization of ribonucleoprotein complex subunits | 0.0001 | Rps5/Hsp90aa1/Hsp90ab1/Rpsa/Rps19/Rps15/Rplp0/Rps27 | 10.50 |
| GO:0007159 | Leukocyte cell-cell adhesion | 0.0001 | Arid1a/Cd83/Hsp90aa1/Smad7/Hsph1/Rps3/Cd37/Ets1/Ripor2/Nfkbid/Ccr7 | 6.28 |
| GO:1901798 | Positive regulation of signal transduction by p53 class mediator | 0.0006 | Ubb/Rps20/Rps7/Rps15 | 30.06 |

*Note: Red indicates genes upregulated in the B[a]P exposure group relative to the B[a]P withdrawal group, whereas green indicates genes downregulated in the B[a]P exposure group relative to the B[a]P withdrawal group.*
