# Supplementary material for: Regulatory effects of smoking cessation on the cellular microenvironment and differentially expressed genes in precancerous lesions of pulmonary nodules in mice based on single-cell RNA sequencing and immune repertoire-sequencing
Source: PLoS One. 2026 Aug 13;21(8):e0356148. doi: 10.1371/journal.pone.0356148 (PMC13472401; doi:10.1371/journal.pone.0356148)
Supplement: Supplementary file 3 [file pone.0356148.s008.docx]

S2 Table

Amino acid types, differentially expressed genes upregulated or downregulated after B[a]P withdrawal, and their functional annotations.

| Group | Differentially Expressed  Amino Acids | Amino Acid Function | Differentially Expressed Genes | Gene Function |
| --- | --- | --- | --- | --- |
| Upregulated  IGH | Y、R、G、S、L | Y enhances antibody affinity  through phosphorylation | IGHV1-39*01、IGHV2-2*02、IGHV5-17*01、  IGHV3-6*01 | Recognizes antigens, mediates adaptive  immune responses |
| Upregulated  IGL/IGK | S、Y | S enhances antibody affinity  through phosphorylation | IGKV10-94*03、IGKV10-96*03、IGKV1-135*01、  IGKV3-10*01、IGKV3-4*01、IGKV4-74*01、  IGKV4-78*01、IGKV8-21*01、IGKV8-24*01 | Provides antigen-binding sites for antibody  light chains |
| Upregulated  TRA | A | A drives initial activation of  immature T cells and  re-stimulation of Tmem cells | TRAV15-2/DV6-2*01、TRAV3-1*01、TRAV8-1*01、  TRAV8-2*01、TRGV1*02、TRAV7-3*01、  TRAV3-3*01、TRAV12D-1*02 | Recognizes antigen peptides-MHC  complexes |
| Upregulated  TRB | A、C、D、K、L、M、P、Q、T | L activates mTORC1 via  SLC7A5 targeting | TRBV1*01、TRBV13-1*01、TRBV13-3*01、  TRBV19*01、TRBV2*01、TRBV3*01、TRBV30*01 | Plays a critical role in activating cytotoxic  T cells |
| Downregulated IGH | W、C、D、V | C regulates the methionine  pathway | IGHV11-2*01 | Somatic hypermutation |
| Downregulated IGL/IGK | M | M produces SAM | IGKV14-126*01、IGKV5-45*01、IGKV6-25*01、  IGKV8-30*01、IGLV2*02 | Affects antibody binding affinity  and specificity |
| Downregulated TRA | H、I、K | H affects T cell functional  responses | TRAV10D*01 | Immune surveillance function |
| Downregulated TRB | F、S | F enhances TCR binding affinity  With antigen peptide-MHC complexes through hydrophobic interactions | TRBV4*01、TRBV12-1*01、TRBV15*01、  TRBV16*01、TRBV20*01 | Highly expressed in the  tumor microenvironment |

*Note: “Upregulated” indicates amino acids or genes enriched in the B[a]P withdrawal group relative to the B[a]P exposure group, whereas “Downregulated” indicates those reduced in the B[a]P withdrawal group. Amino acids are shown using single-letter abbreviations.*
